# Design and Synthesis of Thionated Levofloxacin: Insights into a New Generation of Quinolones with Potential Therapeutic and Analytical Applications

**DOI:** 10.3390/cimb44100316

**Published:** 2022-10-03

**Authors:** Ali I. M. Ibrahim, Hassan Abul-Futouh, Laurance M. S. Bourghli, Mohammad Abu-Sini, Suhair Sunoqrot, Balqis Ikhmais, Vibhu Jha, Qusai Sarayrah, Dina H. Abulebdah, Worood H. Ismail

**Affiliations:** 1 Faculty of Pharmacy, Al-Zaytoonah University of Jordan, P.O. Box 130, Amman 11733, Jordan; 2 Department of Chemistry, Faculty of Science, The Hashemite University, P.O. Box 330127, Zarqa 13133, Jordan; 3 Department of Chemistry and Molecular Biology, University of Gothenburg, 405 30 Göteborg, Sweden

**Keywords:** thionated levofloxacin, antibacterial, molecular docking, anticancer, electronic excitation

## Abstract

Levofloxacin is a widely used fluoroquinolone in several infectious diseases. The structure–activity relationship of levofloxacin has been studied. However, the effect of changing the carbonyl into thiocarbonyl of levofloxacin has not been investigated up to the date of this report. In this work, levofloxacin structure was slightly modified by making a thionated form (compound **3**), which was investigated for its antibacterial activity, biocompatibility, and cytotoxicity, as well as spectroscopic properties. The antibacterial susceptibility testing against five different bacteria showed promising minimum inhibitory concentrations (MICs), particularly against *B. spizizenii* and *E. coli*, with an MIC value of 1.9 µM against both bacteria, and 7.8 µM against *P. mirabilis*. The molecular docking experiment showed similar binding interactions of both levofloxacin and compound **3** with the active site residues of topoisomerase IV. The biocompatibility and cytotoxicity results revealed that compound **3** was more biocompatible with normal cells and more cytotoxic against cancer cells, compared to levofloxacin. Interestingly, compound **3** also showed an excitation profile with a distinctive absorption peak at λ_max_ 404 nm. Overall, our results suggest that the thionation of quinolones may provide a successful approach toward a new generation with enhanced pharmacokinetic and safety profiles and overall activity as potential antibacterial agents.

## 1. Introduction

Quinolones are broad-spectrum antibacterial agents that contain a bicyclic structure of quinolin-4(1*H*)-one nucleus [1]. These agents have therapeutic applications in several infectious diseases, including respiratory infections [2], genitourinary tract infections [3], gastrointestinal tract infections [4], sexually transmitted diseases [5], skin diseases, and soft tissue infections [6]. However, quinolones are rarely used as first-line therapy due to adverse effects and bacterial resistance [7,8,9]. Some studies have linked the occurrence of adverse effects and resistance to the structural features of the quinolone skeleton [10]. However, the quinolone nucleus has also been proven to be essential for antibacterial activity [11]. Therefore, a slight modification of the quinolone nucleus may alter their pharmacokinetic profile without affecting the pharmacological use. Furthermore, quinolones are conjugated aromatic systems and have interesting spectroscopic properties [12]. In fact, several studies have been performed to investigate their analytical properties, separately [13] or in complexation [14], with an effectiveness in several biological applications. In addition, a modification on the quinolone nucleus has been found to have a considerable influence on the wavelength of maximum absorption [15]. Therefore, any structural modification on the quinolone chromophore would be worthy of analytical investigation.

Levofloxacin, the levo isomer of ofloxacin, is a third-generation fluoroquinolone, which has a broad-spectrum antibacterial activity [16]. Levofloxacin inhibits both the bacterial DNA gyrase and topoisomerase IV enzymes, resulting in a bactericidal activity [17]. However, several studies have reported a prevalence of levofloxacin-resistant *Escherichia coli* (*E. coli*) [18,19], *Stenotrophomonas maltophilia* [20], and *Staphylococcus aureus* [21] and a failure in the treatment of several cases of pneumonia [22]. In addition, various studies were performed to improve its bioavailability in ocular [23] and pulmonary [24] tissues. Accordingly, levofloxacin has been considered as an interesting target for research, including drug development and optimization.

**Figure 1:**
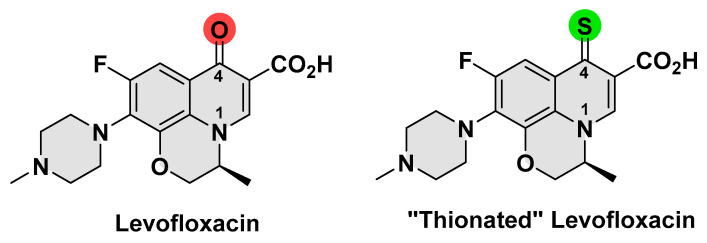
Chemical structure of levofloxacin and the thionated analogue.

This work aimed at structurally modifying levofloxacin via a simple replacement of the carbonyl oxygen at position 4 with a sulfur atom (Figure 1). Similar chemical modification has been found beneficial for some drug classes to solve problems that limit their clinical use [25]. Therefore, the new “thionated” quinolones may show different therapeutic applications (e.g., anticancer [26,27]) alongside the antibacterial activity. Alternatively, the new derivatives may behave as prodrugs that can be activated in vivo through oxidative mechanisms [28], which may minimize the adverse effects or enhance the bacterial sensitivity [28,29]. In addition, the thionated derivatives of quinolones may exhibit interesting excitation/emission profiles that are different from those obtained for quinolones [30], and hence worth investigating.

## 2. Materials and Methods

### 2.1. General

The levofloxacin was a kind gift from Hikma Pharmaceuticals (Amman, Jordan). Lawessen’s reagent was purchased from Sigma-Aldrich (St. Louis, MO, USA). Human dermal fibroblasts (ATCC PCS-201-012) and A549 and H1299 human NSCLC cell lines (LOT numbers 70018877 and 70008730, respectively) were purchased from the American Type Culture Collection (ATCC, Manassas, VA, USA). Phosphate-buffered saline (PBS), Roswell Park Memorial Institute (RPMI)-1640 medium, fetal bovine serum (FBS), and L-glutamine were purchased from Euroclone (Pero, Italy). Dimethyl sulfoxide (DMSO) was purchased from TEDIA (USA). bacterial strains (*Escherichia coli* ATCC 8739, *Pseudomonas aeruginosa* ATCC 9027, *Proteus mirabilis* ATCC 12453, *Bacillus spizizenii* ATCC 6633, *Staphylococcus aureus* ATCC 6538) were reference strains obtained from ATCC. Nutrient agar was purchased from Biolab (Budapest, Hungary). Mueller-Hinton agar medium was purchased from Oxoid (Wade Road, UK). Chemical reactions were monitored by analytical thin layer chromatography using Merck 9385 silica gel 60 F254 aluminum-backed plates through visualizing the spotted plates under ultraviolet (UV) at 254 and 366 nm. Intermediates and final products were purified by column chromatography using silica gel (pore size 60 ÅA, 40–63 µm particle size). ^1^H and ^13^C NMR were analyzed for all intermediates and final products on a Bruker AMX400 (400 MHz) nuclear magnetic resonance spectrometer. Chemical shifts were reported in parts per million (*δ*, ppm) downfield from internal TMS. Coupling constants (J) were expressed in hertz (Hz). High-resolution mass spectra were recorded on a Bruker Impact II HRMS with an ESI source. Chemical structures were drawn using ChemDraw (version 18.0.0231). Melting points were measured with a Gallenkamp melting point apparatus. Dose–response curves were generated using the GraphPad Prism 7 software. Optical densities were then measured on a SynergyHTX^®^ spectrophotometer. IC_50_ values were calculated by non-linear fitting of the experimental data to a sigmoidal plot using GraFit 5.0 (Erithacus software)

### 2.2. Synthesis and Characterization

#### 2.2.1. Methyl (3R)-8-Fluoro-3-methyl-9-(4-methylpiperazin-1-yl)-6-oxo-2,3,3a,6-tetrahydrobenzo[de]chromene-5-carboxylate (Compound **1**)

To 1.0 g of levofloxacin (2.77 mmol) in 100 mL methanol, a few drops of conc. sulfuric acid were added, and the reaction mixture was refluxed using a Dean–Stark apparatus. The consumption of Levofloxacin was monitored by TLC, and, upon completion, excess methanol was distilled off under reduced pressure yielding a crude of yellowish green residue. The crude was dissolved in 20 mL distilled water and treated with approximately 30 mL of saturated sodium carbonate solution, upon which a white precipitate formed, which was filtered and dried, affording 831 mg of the titled product (83% yield). ^1^H NMR (300 MHz, CDCl_3_) δ 8.21 (s, 1H, Vinyl-H), 7.53 (d, *J*_H-F_ = 12.5 Hz, 1H, Ar-H), 4.31 (d, *J* = 10.5 Hz, 2H, O-CH_2_), 3.84 (s, 3H, O-CH_3_), 3.29 (m, 4H, piperazine-CH_2_), 2.51 (m, 4H, piperazine-CH_2_), 2.31 (s, 3H, N-CH_3_), 1.48 (d, *J* = 6.1 Hz, 3H, C-CH_3_). ^13^C NMR (75 MHz, CDCl_3_) δ 171.79, 165.41, 154.74 (d, *J*_C-F_ = 247.0 Hz), 144.38, 138.62 (d, *J*_C-F_ = 6.6 Hz), 130.56 (d, *J*_C-F_ = 14.8 Hz), 122.74, 108.62, 104.77 (d, *J*_C-F_ = 24.1 Hz), 67.14, 54.61, 53.74, 51.06, 49.36, 49.30, 45.21, 17.17. HRMS-ESI (*m/z*): [M + H]^+^ calculated for C_19_H_23_FN_3_O_4_ 376.1594, found 376.1664.

#### 2.2.2. Methyl (3R)-8-Fluoro-3-methyl-9-(4-methylpiperazin-1-yl)-6-thioxo-2,3,3a,6-tetrahydrobenzo[de]chromene-5-carboxylate (Compound **2**)

650 mg of compound 1 (1.73 mmol, 1 eq.) and 770 mg of Lawesson’s reagent (1.90 mmol, 1.1 eq.) were suspended in 50 mL of dry THF. After a few minutes, the suspension turned into a deep orange solution and the suspension was then refluxed under argon for 5 h. THF was then evaporated under reduced pressure yielding a crude, which was purified by column chromatography using methanol/chloroform (1/1), affording 525 mg of the titled product as an orange solid (80% yield). ^1^H NMR (300 MHz, CDCl_3_) δ 7.98 (d, *J*_H-F_ = 12.0 Hz, Ar-H), 7.71 (s, 1H, Vinyl-H), 4.83–4.06 (m, 3H, O-CH_2_, and CH), 3.81 (s, 3H, O-CH_3_), 3.32 (m, 4H, piperazine-CH_2_), 2.54 (m, 4H, piperazine-CH_2_), 2.34 (s, 3H, N-CH_3_), 1.45 (m, 3H, C-CH_3_). ^13^C NMR (75 MHz,) δ 189.11, 166.58, 156.71 (d, *J*_C-F_ = 246.9 Hz), 138.88 (d, *J*_C-F_ = 7.0 Hz), 136.83, 131.77 (d, *J*_C-F_ = 14.1 Hz), 130.11 (d, *J*_C-F_ = 9.8 Hz), 124.53, 120.10, 108.85 (d, *J*_C-F_ = 26.2 Hz), 67.79, 55.56, 52.16, 50.31, 50.26, 46.24, 18.47. HRMS-ESI (*m/z*): [M + H]^+^ calculated for C_19_H_23_FN_3_O_3_S 392.1366, found 392.1450.

#### 2.2.3. (3R)-8-Fluoro-3-methyl-9-(4-methylpiperazin-1-yl)-6-thioxo-2,3,3a,6-tetrahydrobenzo[de]chromene-5-carboxylic Acid (Compound **3**)

To 200 mg of compound 2 (0.51 mmol, 1 eq.) in 30 mL of a THF/water (1/1) mixture, 81.74 mg of NaOH (2.04 mmol, 4 eq.) was added and the reaction mixture was stirred vigorously for 3 h at room temperature. Concentrated HCl was then added dropwise until a yellow precipitate formed, which was filtered and dried, affording 154 mg of the titled product as a yellow solid (77% yield). ^1^H NMR (DMSO-d_6_, 400 MHz) δ 8.83 (s, 1H, Vinyl-H), 8.17 (d, *J*_H-F_ = 13.9 Hz, Ar-H), 4.80–4.20 (m, 3H, O-CH_2_, and CH), 3.45 (m, 4H, piperazine-CH_2_), 2.62 (m, 4H, piperazine-CH_2_), 2.40 (s, 3H, N-CH_3_), 1.63 (m, 3H, C-CH_3_). ^13^C NMR (101 MHz, CDCl_3_) δ 187.16, 167.73, 157.09 (d, *J*_C-F_ = 249.5 Hz), 141.41, 138.71 (d, *J*_C-F_ = 7.2 Hz), 133.06 (d, *J*_C-F_ = 14.5 Hz), 128.79 (d, *J*_C-F_ = 10.1 Hz), 120.29, 109.22 (d, *J*_C-F_ = 26.1 Hz), 68.00, 56.65, 55.60, 50.44, 46.25, 29.82, 18.63. HRMS-ESI (*m/z*): [M + H]^+^ calculated for C_18_H_20_FN_3_O_3_S 377.1209, found 377.1219.

### 2.3. Microbiological Procedure

#### 2.3.1. Microorganisms

The microorganisms used in this study consisted of five bacterial strains including three Gram-negative bacteria (*Escherichia coli*, *Pseudomonas aeruginosa*, and *Proteus mirabilis*) and two Gram-positive bacteria (*Bacillus spizizenii* and *Staphylococcus aureus*). The bacterial strains were grown at 37 °C and maintained on nutrient agar.

#### 2.3.2. Well Diffusion Methods

Compounds **1**, **2,** and **3** were tested in vitro for their antimicrobial activity against Gram-positive and Gram-negative bacteria by the Kirby–Bauer method [31]. The media used for bacteria was Mueller-Hinton agar medium. Bacterial colonies were prepared in 5 mL phosphate-buffered saline (0.5 McFarland standards), then 100 µL of bacterial culture was inoculated on fresh Mueller-Hinton agar using a cotton swab. Next, wells were bored on the Muller-Hinton agar plates with the help of a sterilized borer. Then, each well was filled with 50 μL of the tested compounds with serial concentrations (2.0, 0.5, 0.25, 0.125, 0.062, 0.031, 0.015, 0.007, 0.0039, 0.0019, and 0.0009 mM), with DMSO concentrations of 5.0%, 1.25%, 0.625%, 0.31%, 0.15%, 0.07%, 0.03%, 0.01%, 0.009%, 0.004%, 0.002%, respectively, in each sample. Levofloxacin was used a as positive control, whereas a concentration of 5% of DMSO was used as a negative control. The inoculated plates with different pathogenic bacteria were incubated at 37 °C for 18 to 24 h. Antimicrobial activity was evaluated by measuring the inhibition zone against tested bacteria. All tests were repeated three times and an average of the triplicate was considered.

#### 2.3.3. Minimum Inhibitory Concentration (MIC)

A broth microdilution method was employed to determine the minimum inhibitory concentration (MIC) [32]. The microorganisms used in this test were five bacteria (*Escherichia coli*, *Pseudomonas aeruginosa*, *Proteus mirabilis*, *Bacillus spizizenii*, *and Staphylococcus aureus*). Bacterial suspensions were prepared to 0.5 McFarland standards. A serial doubling dilution of the compounds was prepared in a 96-well microtiter plate. A Mueller-Hinton broth was used as a diluent. Stock solutions with concentrations of 1 mM in DMSO were prepared for the tested compounds. The two-fold serial dilution was performed in this experiment to get the concentration range of the derived compounds (0.5–0.00097 mM) and antibiotic control (0.5–0.00097 mM). For every experiment, a sterility check (medium), negative control (medium), and positive control (medium and inoculum) were included, the 96-micro well plates were prepared by dispensing 100 μL of the appropriate medium into each well, then 100 μL of the tested compounds were added into the first well and serially diluted to 10 wells. Then, 100 μL of bacterial inoculums were added to each well. The content of each well was mixed thoroughly with a multi-channel pipette, and the microwell plates were covered with the sterile sealer and incubated at 37 °C for 24 h. Levofloxacin was used as a positive control. The absorbance of the wells was read at 570 nm using a microtiter plate reader after incubation. The percentages of inhibition of bacteria growth were calculated by using the following formula [3]:

$$\mathrm{Percentage}\;\mathrm{of}\;\mathrm{inhibition}\;(\%)=\frac{1-\left(\mathrm{OD}\;\mathrm{test}\;\mathrm{well}–\mathrm{OD}\;\mathrm{corresponding}\;\mathrm{negative}\;\mathrm{control}\;\mathrm{well}\right)}{\left(\mathrm{OD}\;\mathrm{viability}\;\mathrm{control}\;\mathrm{well}–\mathrm{OD}\;\mathrm{broth}\;\mathrm{only}\;\mathrm{well}\right)}\times 100\%$$

The MIC assay was repeated three times and values were determined as an average of triplicate.

#### 2.3.4. Minimum Bactericidal Concentrations (MBC)

After the MIC determination of the compounds, 10 μL from all the wells that showed no visible bacterial growth (no turbidity) were seeded on Muller-Hinton agar plates and incubated for 24 h at 37 °C. When 99.9% of the bacterial population is killed at the lowest concentration of an antimicrobial agent, it is termed as the MBC endpoint. This assay was performed by observing pre- and post-incubated agar plates for the presence or absence of bacteria [33].

### 2.4. Biocompatibility Assay

Biocompatibility of the compounds was evaluated in human dermal fibroblasts (ATCC) grown in Iscove’s modified Dulbecco’s medium (IMDM; Eurobio Scientific, Les Ulis, France) supplemented with 10% heat-inactivated fetal bovine serum (FBS) (EuroClone, Italy), 1% penicillin-streptomycin (10,000 U/mL each, Gibco, Thermofisher Scientific, Waltham, MA, USA) and 2 mM L-glutamine (EuroClone, Italy). Cells were maintained at 37 °C in a humidified atmosphere with 5% CO_2_. For the experiment, cells (*n* = 5) were seeded in 96-well plates at a density of 7 × 10^3^ cells per well overnight. The next day, cells were treated with different concentrations of the compounds (0.1, 1, 10, 100, and 200 µM diluted in complete culture medium from 20 mM stock solutions in DMSO) for 48 h. At the end of the incubation period, the media were removed and 100 µL of fresh medium containing 0.5 mg/mL 3-(4,5-dimethylthiazol-2-yl)-2,5-diphenyltetrazolium bromide (MTT, Sigma-Aldrich, USA) was added to each well. The plates were incubated for 3 h, then the media were carefully removed, and the formazan crystals were dissolved in 100 µL DMSO with gentle mixing. The optical density of each well was measured at 540 nm using a Synergy HTX Multi-Mode Reader (BioTek, Winooski, VT, USA). Cell viability % was expressed relative to control wells, which were treated with complete medium only.

### 2.5. Cytotoxicity Assay

For the cytotoxicity assay, A549 and H1299 human NSCLC cell lines were cultured in RPMI-1640 medium, supplemented with 10% (*v*/*v*) heat-inactivated FBS and 2 mM L-glutamine. The cells were maintained in 5% CO_2_ humidified incubator at 37 °C. For the experiment, A549 and H1299 cells (*n* = 3) were seeded into 96-well plates at densities of 750 and 500 cells per well, respectively, and left overnight. Cells were then treated with the compounds at various concentrations ranging between 0.01 and 200 µM for 96 h. The % of surviving cells was then determined using the MTT assay as described above. Optical densities were then measured on a Synergy HTX Multi-Mode Reader at 540 nm and then analyzed using Gen5 Software package. The results were used to calculate the surviving % of cells relative to solvent-only controls. Dose–response curves were generated using the GraphPad Prism 9.3.1 software, and non-linear regression analysis was used to fit the data. IC_50_ values (defined as the concentration of drug required to decrease cell survival by 50% relative to controls) were determined from these curves.

### 2.6. Molecular Modeling

#### 2.6.1. Protein and Ligand Preparation

The X-ray structure of the Quinolone-(Levofloxacin)-DNA cleavage complex of type IV topoisomerase from *S. pneumoniae* (PDB code: 3RAE [34]) was downloaded from the Protein Data Bank. The resolution of the X-ray structures was found to be 2.90 Å. The X-ray structure was primarily selected, after satisfying the X-ray diffraction resolution of not more than 3.0 Å (higher values are associated with poor quality). The co-crystallized ligands, ions, and water molecules were removed from the X-ray complexes and H-bonds, and missing residues were added to the protein with the aid of the protein preparation wizard of Maestro. The synthesized compound was drawn using the Build panel of Maestro and subjected to the LigPrep tool interfaced with the Maestro module of the Schrödinger suite. The 3D structure and ionization states at pH 7.0 ± 2.0 of the synthesized compounds were generated and geometrically minimized using an OPLS3e force field.

#### 2.6.2. Molecular Docking

Docking calculations were carried out on the X-ray structure of a Quinolone-(Levofloxacin)-DNA cleavage complex of type IV topoisomerase from *S. pneumoniae* (PDB code: 3RAE). Glide 5.0 with the standard precision (SP) method was used for docking of the synthesized compound on the above-mentioned X-ray structure.

#### 2.6.3. GLIDE 5.0 

The binding site was defined by a rectangular box of 10 Å in the x, y, and z directions cantered on the ligand. The possibility of imposing a maximum number of atoms a ligand may have if it was to be docked was deactivated, so that all the ligands were docked independently from the number of their atoms, whereas the GLIDE defaults were used for all other parameters [35]. The GlideScore fitness function is based on Chemscore but includes a steric-clash term and adds buried polar terms to penalize electrostatic mismatches and modifications on other secondary terms. The docking analyses were carried out using the standard precision (SP) method. A total of 50 docking solutions were generated for the synthesized ligand and the top-ranked docking pose was considered as the final pose.

## 3. Results and Discussion

### 3.1. Synthesis and Characterization of Thionated Levofloxacin

**Scheme 1:**
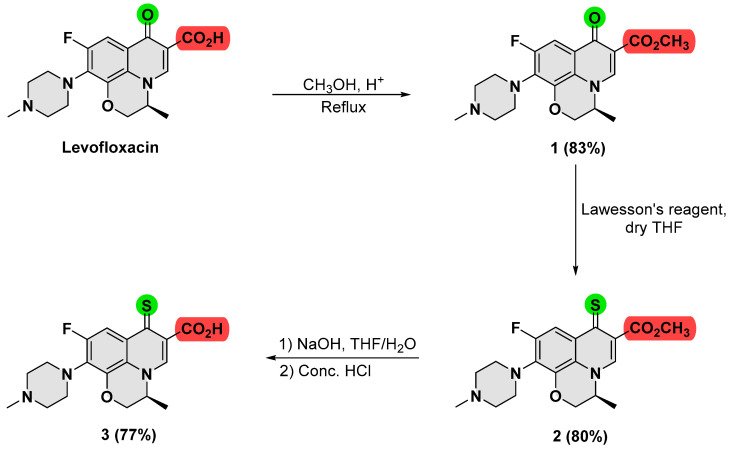
Synthetic pathways of obtaining compound 3.

The thionated levofloxacin, compound **3**, was synthesized from levofloxacin through several steps, as shown in Scheme 1.

As a first step, levofloxacin was converted into its ester form (compound **1**), in which a catalytic amount of sulfuric acid and a Dean–Stark apparatus were used to force the reaction towards completion. ^1^H-NMR, ^13^C{^1^H}-NMR, and HRMS (Supplementary Figures S1, S2, and S3, respectively) confirmed the obtainment of compound **1**, in which the absence of a carboxylic acid peak in the ^1^H-NMR spectrum together with the appearance of singlet peaks at δ3.84 and δ53.7 for ^1^H and ^13^C-NMR, respectively, confirmed the formation of the methyl ester. Compound **1** was then thionated using Lawessen’s reagent, whereby the carbonyl oxygen from the ketone group was effectively substituted by Lawessen’s reagent and chemically changed to sulfur, affording compound **2** as an orange solid, with a relatively good yield (80%). The structure of this product was confirmed by ^13^C{^1^H}-NMR (Supplementary Figure S5), which showed a significant shifting from δ171.8 (the starting carbonyl carbon) to a higher chemical shift value (δ189.1) for the thiocarbonyl carbon. In addition, the HRMS spectrum (Supplementary Figure S6) showed an ion peak MH^+^ at *m/z* 392.145, which confirmed the chemical formula of compound **2** (C_19_H_22_FN_3_O_3_S). Compound **3** was then synthesized through ester hydrolysis of compound **2** using sodium hydroxide in a mixture of water/THF, followed by acidic neutralization. The chemical structure of compound **3** was characterized by ^1^H-NMR, ^13^C{^1^H)-NMR, and X-ray crystallography (Supplementary Figures S7, S8 and S9, respectively), in which the appearance of a proton peak at δ9.19 and disappearance of the methyl peak at δ3.84 confirmed the successful hydrolysis reaction. Regarding X-ray, the crystallization of **3** unfortunately provided low-quality crystals that resulted only in a structural motif of compound **3** (Figure S9).

The synthetic procedures, purification, and characterization for all synthetic compounds were provided in detail previously at the experimental section.

### 3.2. Antimicrobial Activity

#### 3.2.1. Well Diffusion Method

The growth inhibition zones were measured for the synthesized compounds (**1**, **2,** and **3**) as a preliminary screening of their antibacterial activity on five different bacterial strains: two Gram-positive bacteria (*Bacillus spizizenii* and *Staphylococcus aureus*) and three Gram-negative bacteria (*E. coli*, *Pseudomonas aeruginosa*, and *Proteus mirabilis*). For each compound, serial concentrations of 2.0, 0.5, 0.25, 0.125, 0.062, 0.031, 0.015, 0.007, 0.0039, 0.0019, and 0.0009 mM was prepared, with final DMSO concentrations of 5.0%, 1.25%, 0.625%, 0.31%, 0.15%, 0.07%, 0.03%, 0.01%, 0.009%, 0.004%, and 0.002%, respectively, in each sample. The inhibition zones produced by 2.0 mM concentrations of the synthesized compounds were compared with levofloxacin as a positive control as shown in Table 1, and for all tested concentrations the results are shown in Supplementary Table S1 and Figure S10.

The results showed that compound **3** provided the highest inhibition of the bacterial growth among the synthesized compounds in all tested bacteria; however, it was less active than levofloxacin. These preliminary results were further verified via the dilution method [36].

#### 3.2.2. Dilution Method

The five selected bacteria were treated by 15 serially diluted solutions of each synthesized compound to accurately measure the minimum inhibitory concentration (MIC) and minimum bactericidal concentration (MBC) of each compound, with results shown in Table 2. The detailed results of % inhibitions in bacterial growth for each compound at a wide range of concentrations were indicated in Supplementary Figure S11. The obtained results were found to be correlated with the diffusion method results, in which compound **3** showed the highest antibacterial activity among the synthesized compounds, albeit it was lower than levofloxacin. In particular, compound **3** showed a promising antibacterial activity with MIC values of 1.9 µM against both *B. spizizenii* and *E. coli*, and 7.8 µM against *P. mirabilis*, while the MBC values were found to be 3.9 µM, 15.6 µM, and 31.25 µM, respectively. It is important to highlight that compound **3** has a free carboxylic acid group, whereas it is an ester in compounds **1** and **2**. Therefore, because both **2** and **3** were thionated analogues, the achieved results were found in agreement with the fact that the carboxylic acid moiety of quinolones is part of the pharmacophore and essential for the antibacterial activity [37].

From the bacterial susceptibility tests, compound **3** showed a lower antibacterial activity than levofloxacin against all tested bacteria, which indicated that replacing the oxygen by sulfur atom did not provide an enhanced in vitro antibacterial activity. However, this observation was not seen when comparing the antibacterial activity of compound **1** (has a carbonyl group) with compound **2** (has a thiocarbonyl group), where both compounds showed almost similar antibacterial activity against the tested bacteria, which perhaps indicated the importance of the free carboxylic acid moiety for activity.

From a computational perspective, the theoretical LogP value of compound **3** was measured and found to be 1.90, which was higher than levofloxacin (LogP = 1.35), which indicated that replacing the carbonyl oxygen in levofloxacin with a sulfur atom increases the lipophilicity. This may exhibit an enhanced antibacterial activity in vivo assuming an improved pharmacokinetic profile. In addition, there is a high potential for compound **3** to act as a prodrug, which may be expectedly activated either in vivo or by bacterial enzymes, minimizing levofloxacin’s side effects. Therefore, the biocompatibility assay and toxicity evaluation of compound **3** would provide an insight into considering this agent for future investigations.

### 3.3. Biocompatibility and Cytotoxicity Assays

**Figure 2:**
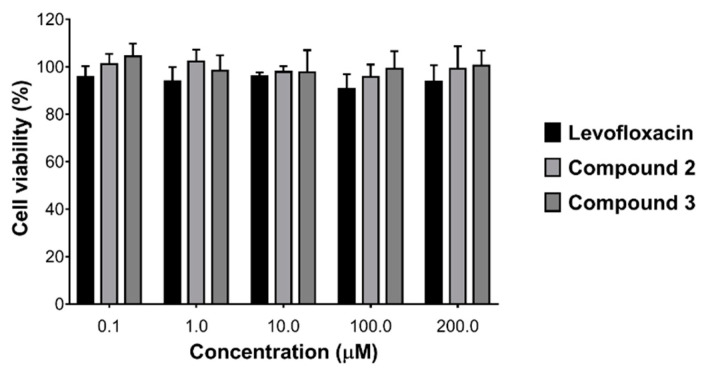
Cell viability of human dermal fibroblasts treated with various concentrations of compounds **2** and **3** and levofloxacin for 48 h.

The biocompatibility of various concentrations of compounds **2** and **3** was evaluated in human dermal fibroblasts as a model for normal cells, and the results were compared with those obtained for levofloxacin. As shown in Figure 2, the results indicated no toxicity for compounds **2** and **3** in all tested concentrations, which were also found slightly less toxic than levofloxacin. This difference, however insignificant, may suggest that compound **3** could demonstrate a superior therapeutic profile compared to levofloxacin.

**Figure 3:**
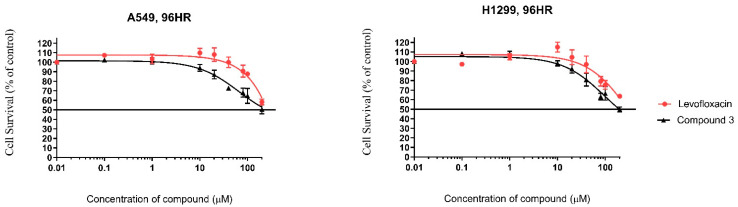
Cell survival percentages for A549 and H1299 cell lines after 96 h treatment with compound **3** and levofloxacin.

As for the cytotoxicity assay, the potential antiproliferative activity of compound **3** was investigated against two non-small cell lung cancer cell lines (NSCLC): A549 and H1299. These cell lines were randomly selected as examples of cancer cells to provide an idea about the potential anticancer activity of the thionated levofloxacin. As also shown in Figure 3, compound **3** exhibited dose-dependent cytotoxicity against both cell lines, with a noticeable increase in cytotoxicity compared to levofloxacin, particularly at high concentrations. The results were further evaluated by calculating the IC_50_ values, which are depicted in Table 3. Notably, compound **3** exhibited lower IC_50_ values against both cancer cell lines compared to levofloxacin. This finding indicates a potential therapeutic utility for this derivative as an anticancer agent and provides clues into further structural optimization of quinolones for cancer therapy.

Taken together, both biocompatibility and cytotoxicity results suggest that compound **3** may be considered as a potential therapeutic agent in infectious diseases, for both cancer and non-cancer patients.

### 3.4. Molecular Docking

**Figure 4:**
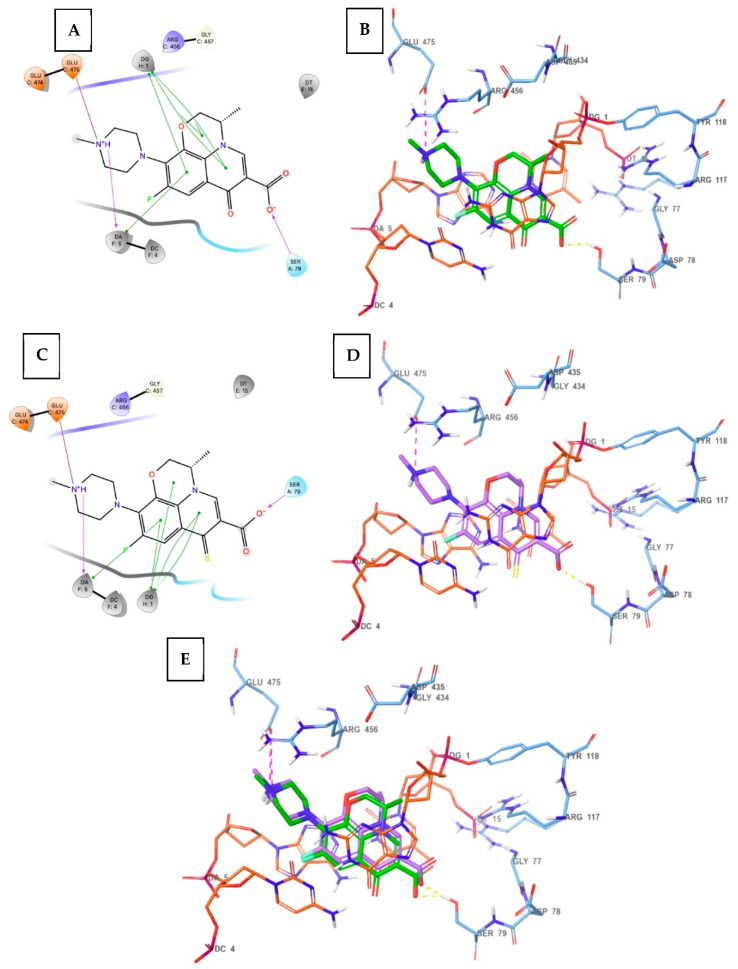
2D (panel (**A**)) and 3D (panel (**B**)) crystallographic binding mode of levofloxacin (green) within the binding site of *S. pneumoniae*; 2D (panel (**C**)) and 3D (panel (**D**)) docking pose of compound **3** (purple) within the binding site; and (panel (**E**)) superposition of levofloxacin and compound **3** within the binding site (residue atoms view).

The X-ray structure of Quinolone (co-crystallized with Levofloxacin) and DNA cleavage complex of topoisomerase IV from *S. pneumoniae* (PDB ID: 3RAE [34]) were employed in docking studies. Compound **3** was docked into the active site of this complex to predict its binding mode and compare with the crystallographic orientation of levofloxacin. As illustrated in the following 2D and 3D figures (Figure 4), the tricyclic moiety of both levofloxacin and compound **3** formed strong face-to-face pi–pi stacking with the guanine nucleus of DG1 (deoxyguanosine nucleotide). In addition, the adenine nucleus of D5 (deoxyadenosine nucleotide) is also involved in the stacking interactions with the tricyclic moiety of both levofloxacin and compound **3**. The carboxy group of both levofloxacin and compound **3** established an important H-bond contact with Ser79 from the enzyme binding site, while the terminal piperazine sidechain of both levofloxacin and compound **3** constituted H-bond interactions with Glu475 from the topoisomerase IV binding site and DA5. The replacement of oxo of levofloxacin with sulphur in compound **3** did not produce any significant difference, as both oxo (levofloxacin) and sulphur (compound **3**) were not involved in the intermolecular interactions. However, the binding mode was very well reproduced by compound **3** as compared to the crystallographic binding mode of levofloxacin, meeting the above-described intermolecular interactions, significantly contributing to the binding affinity for the topoisomerase-DNA complex of *S. pneumoniae*.

### 3.5. Spectroscopic Analysis

**Figure 5:**
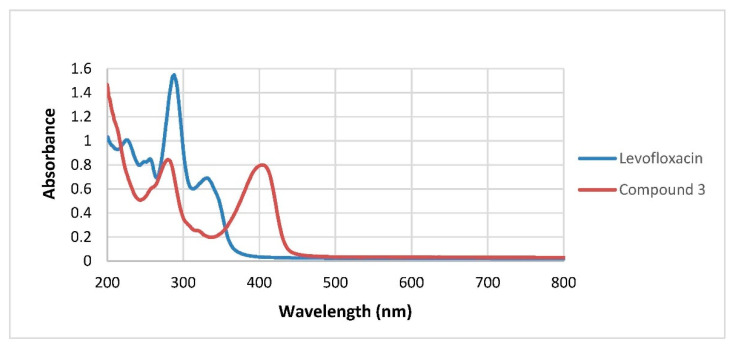
The excitation spectra of both levofloxacin and compound **3**.

Carbonyl and thiocarbonyl compounds have been found to have different electrochemical properties, particularly when they are part of a conjugated system [38]. Therefore, the excitation profile for compound **3** (the thionated levofloxacin) was measured and compared with parent levofloxacin, as shown in Figure 5. Interestingly, both compounds showed markedly different excitation behaviors, particularly at around 360–450 nm, in which compound **3** exposed a distinctive absorption peak at λ_max_ = 404 nm, whereas levofloxacin almost did not absorb energy at this wavelength. This revealed the considerable effect that occurred because of this chemical modification. These results provided a possible method for future identification and quantification purposes, overcoming any overlapping with the levofloxacin or background interference.

## 4. Conclusions

Levofloxacin was structurally modified by changing the carbonyl oxygen to a sulfur atom, producing thionated levofloxacin (compound **3**). This compound was tested for its antibacterial activity and found to have promising results in both well diffusion and serial dilution antibacterial susceptibility test methods, with an MIC value of 1.9µM against both *B. spizizenii* and *E. coli*. In addition, compound **3** was investigated for its biocompatibility and cytotoxicity in human dermal fibroblasts and non-small lung cancer cells, respectively, and found to be safe against the tested normal cells and more cytotoxic against the tested cancer cells (IC_50_ 180 ± 20 µM and 173.3 ± 16.7 µM against A549 and H1299 cells, respectively) compared to levofloxacin (IC_50_ > 200 µM against either cell lines). The analytical investigation of compound **3** showed a remarkable alteration in the absorption curve showing a characteristic absorption peak at λ_max_ 404 nm, compared to the parent levofloxacin. This modified spectrum may help in future work, where identification and quantification experiments are required to assess the compound’s therapeutic and pharmacokinetic profiles. In addition, the thionated levofloxacin may be considered as a novel proligand for complexation reactions with several metals, such as ruthenium, which will be described in a subsequent paper. This modification provides important insights into the structural optimization of quinolones to enhance their antibacterial and anticancer cytotoxicity, as well as spectroscopic properties.

## Figures and Tables

**Table 1:** The zones of inhibition (mm) for 2.0 mM of the synthesized compounds and levofloxacin against five different bacterial strains.

Compound	*B. spizizenii*	*S. aureus*	*E. coli*	*P. aeruginosa*	*P. mirabilis*
**1**	19	12	19	NZ	20
**2**	20	11	20	NZ	22
**3**	26	21	23	13	25
Levofloxacin	35	32	26	29	29
DMSO	NZ	NZ	NZ	NZ	NZ

DMSO: negative control, levofloxacin: positive control, NZ: no zone of inhibition.

**Table 2:** The minimum inhibitory concentration (MIC) and minimum bactericidal concentration (MBC) results for the synthesized compounds compared to levofloxacin against five different bacteria. Concentrations were measured in µM.

Compound	*B. spizizenii*		*S. aureus*		*E. coli*		*P. aeruginosa*		*P. mirabilis*	
	MIC	MBC	MIC	MBC	MIC	MBC	MIC	MBC	MIC	MBC
**1**	3.9	23.4	125	500	7.8	125	NT	NT	15.6	250
**2**	3.9	21.4	250	>500	7.8	125	NT	NT	15.6	250
**3**	1.9	3.9	62.5	250	1.9	15.6	31.25	125	7.8	31.25
Levofloxacin	0.06	0.4	0.9	3.9	0.24	3.9	0.24	1.9	0.48	7.8

NT: not tested.

**Table 3:** Cytotoxicity against A549 and H1299 cells following 96 h exposure.

Compound	IC_50_ (µM) at 96 h	
	A549	H1299
**3**	180 ± 20	173.3 ± 16.7
Levofloxacin	>200	>200

## Data Availability

Not applicable.

## Supplementary Materials

The following are available online at https://www.mdpi.com/article/10.3390/cimb44100316/s1, Figure S1: The 1H NMR spectrum for compound **1**, Figure S2: The 13C NMR spectrum for compound **1**, Figure S3: HRMS spectrum for compound **1**, Figure S4: The 1H NMR spectrum for compound **2**, Figure S5: The 13C NMR spectrum for compound **2**, Figure S6: HRMS spectrum for compound **2**, Figure S7: The 1H NMR spectrum for compound **3**, Figure S8: The 13C NMR spectrum for compound **3**, Figure S9: Motif structure of compound **3**. Due to the poor quality of the crystal, we could not deposit this compound in the Cambridge data base, Figure S10: The zones of inhibition for the tested compounds at stock solutions and 0.5 (1), 0.25 (2), 0.125 (3), 0.062 (4), 0.031 (5), 0.015 (6), 0.007 (7), 0.0039 (8), 0.0019 (9) and 0.0009 (10) mM concentration measured by disk diffusion method against: A. *Bacillus spizizenii*, B. *Staphylococcus aureus*, C. *Escherichia coli* (*E. coli*), D. *Pseudomonas aeruginosa*, E. *Proteus mirabilis*. Levofloxacin was used as the positive control (+VE), DMSO (5%) as negative control (-VE), Figure S11: The percentage of inhibition of several bacterial strains by compounds **3** (orange), **2** (gray), **1** (yellow) and levofloxacin (blue), Table S1: The zones of inhibition (mm) of the synthesized compounds and levofloxacin against five different bacterial strains. DMSO was used as a negative control.
