# Supplementary material for: Design and Synthesis of Thionated Levofloxacin: Insights into a New Generation of Quinolones with Potential Therapeutic and Analytical Applications
Source: Curr Issues Mol Biol. 2022 Oct 3;44(10):4626–38. doi: 10.3390/cimb44100316 (PMC9600924; doi:10.3390/cimb44100316)
Supplement: Supplementary file 1 [file cimb-44-00316-s001.zip › cimb-1909752-supplementary.pdf]

Supporting Information

Design and Synthesis of Thionated Levofloxacin: Insights into a New Generation of Quinolones with Potential Therapeutic and Analytical Applications

Ali I. M. Ibrahim [1*], Hassan Abul-Futouh [2*], Laurance M. S. Bourghli [1], Mohammad Abu-Sini [1], Suhair Sunoqrot [1], Balqis Ikhmais [1], Vibhu Jha [3], Qusai Sarayrah [1], Dina H. Abulebdah [1] and Worood H. Ismail [1]

[1] Faculty of Pharmacy, Al-Zaytoonah University of Jordan, P.O.Box 130, Amman 11733, Jordan

[2] Department of Chemistry, Faculty of Science, The Hashemite University, P.O. Box 330127, Zarqa 13133, Jordan

[3] Department of Chemistry and Molecular Biology, University of Gothenburg, 405 30 Göteborg, Sweden

* Correspondence: A.I.M.I. a.ibrahim@zuj.edu.jo; H.A.F h.abulfutouh@hu.edu.jo.

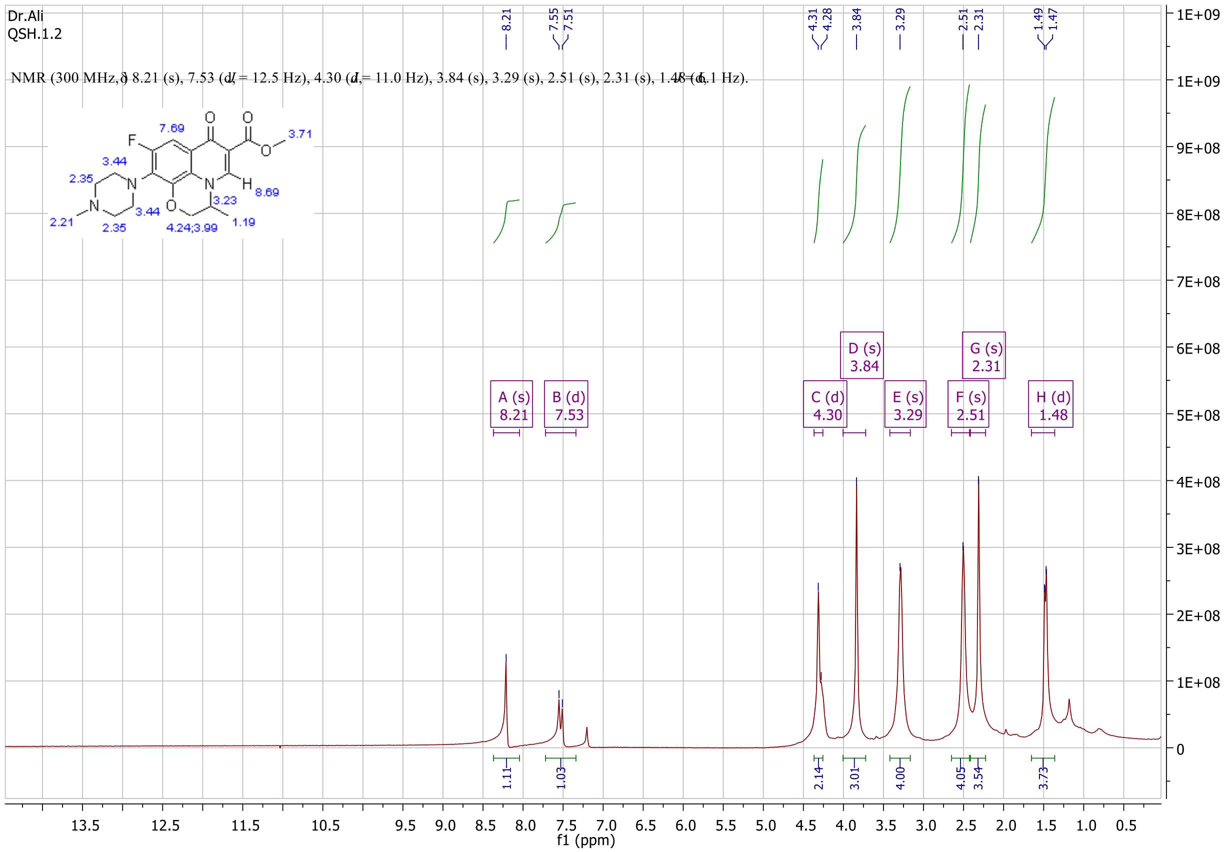

Figure S1. The ¹H NMR spectrum for compound 1.

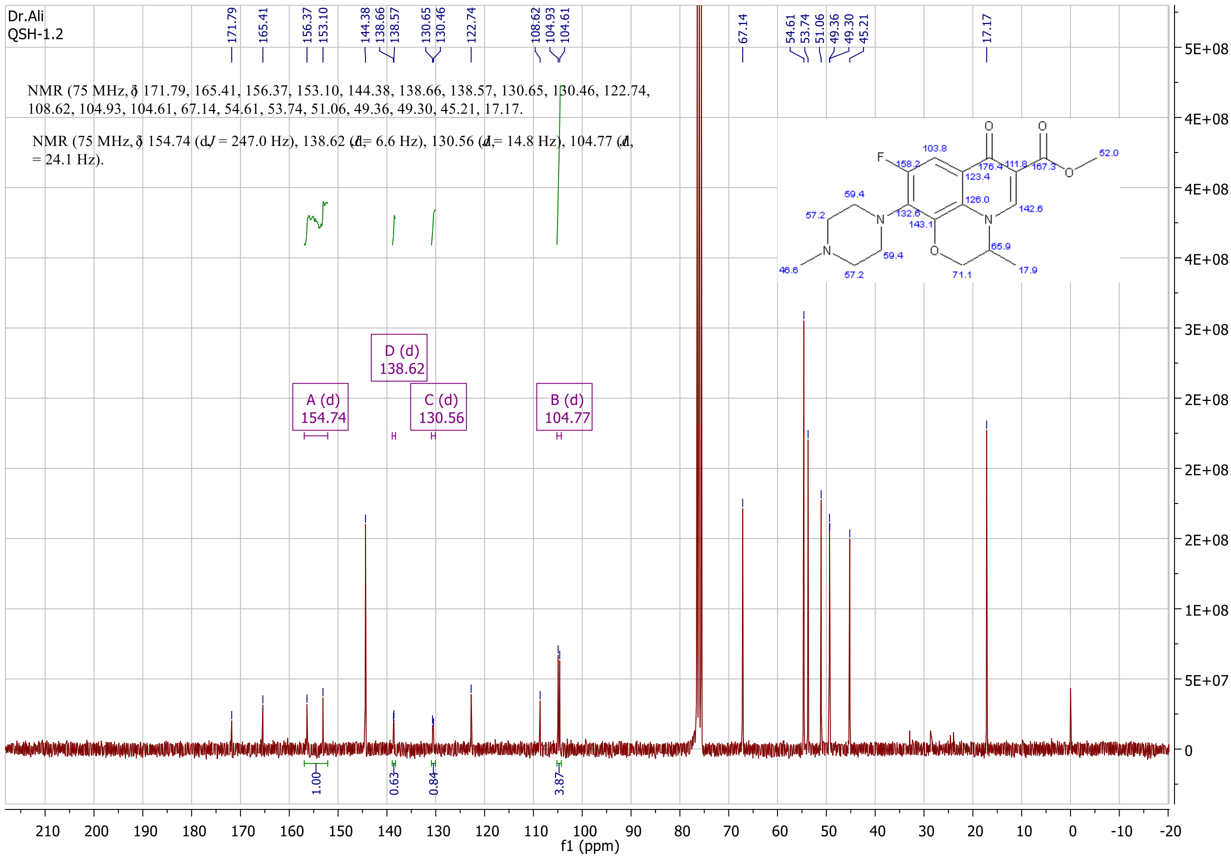

Figure S2. The ^{13}C NMR spectrum for compound 1.

Compound Spectrum List Report

Analysis Info

Analysis Name	\\Esitof\d\Data\March_29_2021\March_25_2021_Dr Luraans QSH.1.2_89_1_1874.d	Acquisition Date	3/29/2021 4:27:36 PM
Method	TargetScreener_impact-II_POS_bbCID.m	Operator	Demo User
Sample Name	March_25_2021_Dr Luraans QSH.1.2	Instrument	impact II 1825265.10265
Comment			

Acquisition Parameter

Source Type	ESI	Ion Polarity	Positive	Set Nebulizer	2.0 Bar
Focus	Active	Set Capillary	2500 V	Set Dry Heater	200 °C
Scan Begin	30 m/z	Set End Plate Offset	-500 V	Set Dry Gas	8.0 l/min
Scan End	1000 m/z	Set Collision Cell RF	1000.0 Vpp	Set Divert Valve	Waste

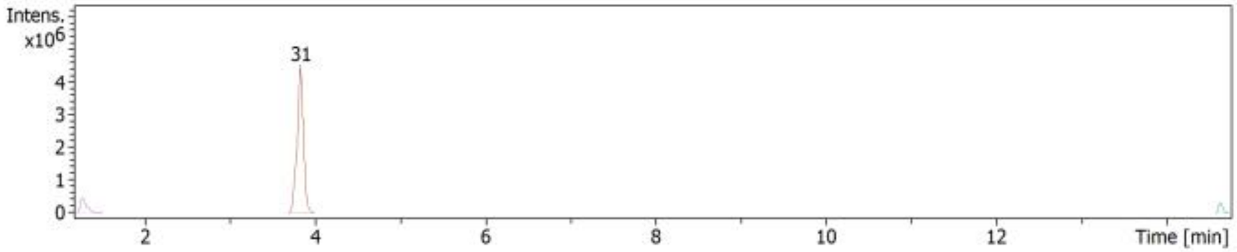

#	RT [min]	Area	Int. Type	I	S/N	Trace	Max. m/z	FWHM [min]
31	3.8	23066640	Dissect	4489827	374.6	Dissect Cmpd 31, Dissect, 3.8 min	376.1664	0.1

C19H22N3O4F

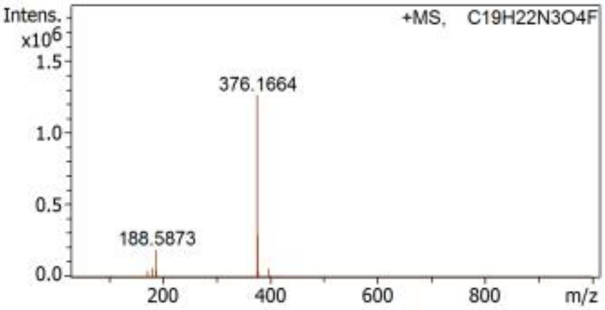

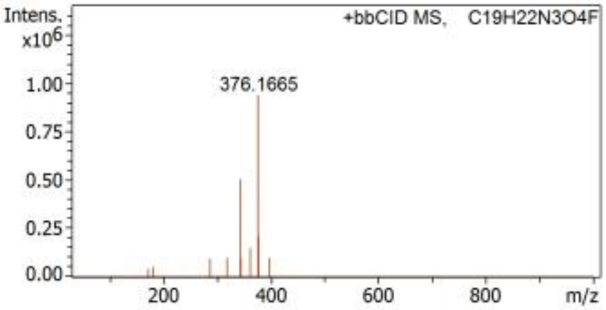

#	m/z	Res.	S/N	I	I %	FWHM
1	172.5742	29526	32.8	44159	3.5	0.0058
2	181.5796	30566	50.8	68410	5.4	0.0059
3	182.0811	29441	9.6	12868	1.0	0.0062
4	188.5873	30915	141.5	190624	15.1	0.0061
5	189.0889	30031	31.1	41938	3.3	0.0063
6	376.1664	41732	935.9	1260596	100.0	0.0090
7	377.1696	37826	217.4	292866	23.2	0.0100
8	378.1722	32345	28.8	38749	3.1	0.0117
9	398.1483	34697	47.2	63606	5.0	0.0115
10	399.1514	32771	10.2	13673	1.1	0.0122

#	m/z	Res.	S/N	I	I %	FWHM
1	172.5742	30068	45.2	46180	4.9	0.0057
2	181.5796	30284	53.7	54845	5.8	0.0060
3	287.0825	34196	94.3	96426	10.3	0.0084
4	319.1086	35313	97.8	99956	10.7	0.0090
5	344.1402	39657	494.2	505094	53.9	0.0087
6	345.1434	34998	98.8	100934	10.8	0.0099
7	362.1511	34487	147.6	150856	16.1	0.0105
8	376.1665	41211	917.3	937548	100.0	0.0091
9	377.1697	37480	208.5	213094	22.7	0.0101
10	398.1484	36106	97.3	99481	10.6	0.0110

Figure S3. HRMS spectrum for compound 1.

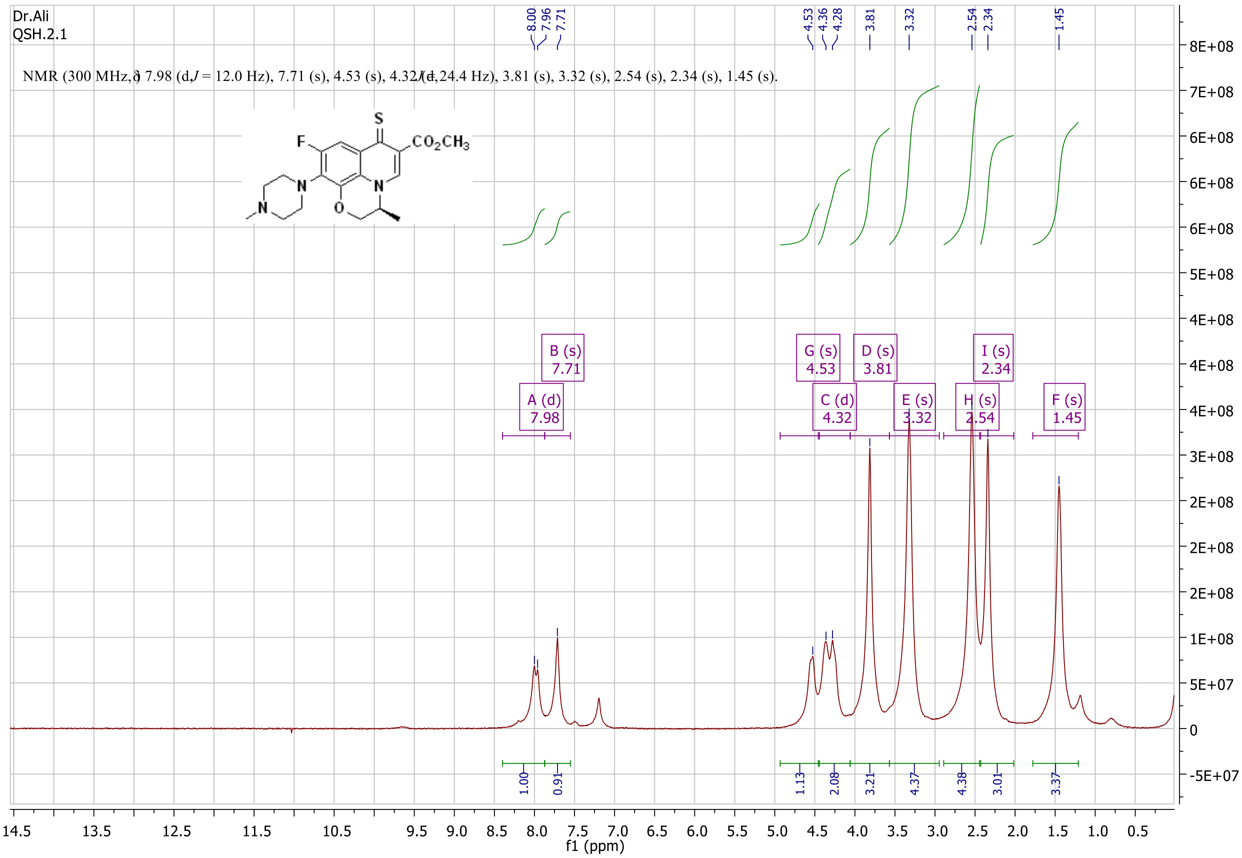

Figure S4. The ^{1}H NMR spectrum for compound 2.

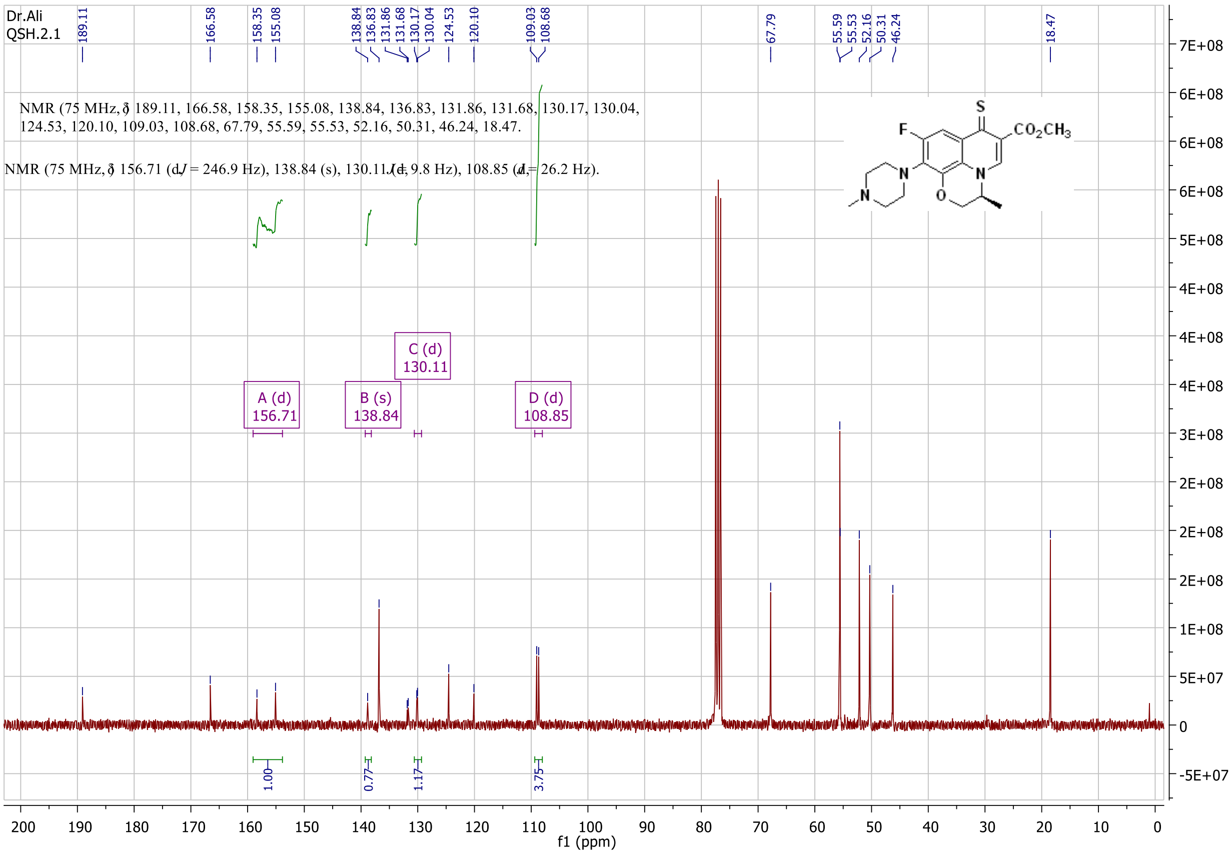

Figure S5. The ^{13}C NMR spectrum for compound 2.

Compound Spectrum List Report

Analysis Info

		Acquisition Date	3/30/2021 2:38:06 PM
Analysis Name	\\Esitof\d\Data\March_29_2021\March_30_2021_Dr Ali QSH.2.1_58_1_1891.d		
Method	TargetScreener_impact-II_POS_bbCID.m	Operator	Demo User
Sample Name	March_30_2021_Dr Ali QSH.2.1	Instrument	impact II 1825265.10265
Comment			

Acquisition Parameter

Source Type	ESI	Ion Polarity	Positive	Set Nebulizer	2.0 Bar
Focus	Active	Set Capillary	2500 V	Set Dry Heater	200 °C
Scan Begin	30 m/z	Set End Plate Offset	-500 V	Set Dry Gas	8.0 l/min
Scan End	1000 m/z	Set Collision Cell RF	1000.0 Vpp	Set Divert Valve	Waste

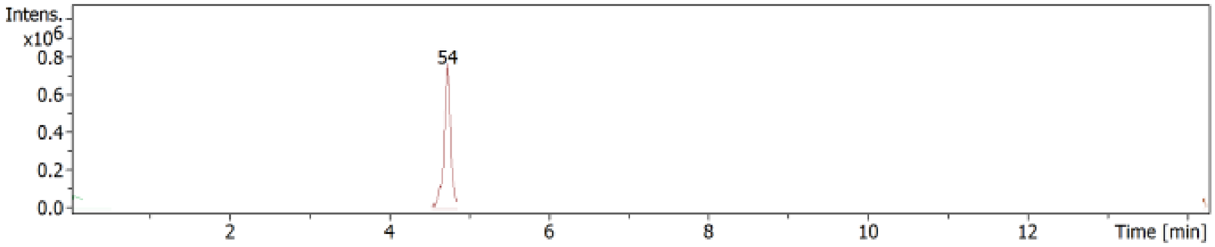

#	RT [min]	Area	Int. Type	I	S/N	Trace	Max. m/z	FWHM [min]
54	4.7	4361464	Dissect	738272	260.5	Dissect Cmpd 54, Dissect, 4.7 min	392.1450	0.1

C19H22FN3O3S

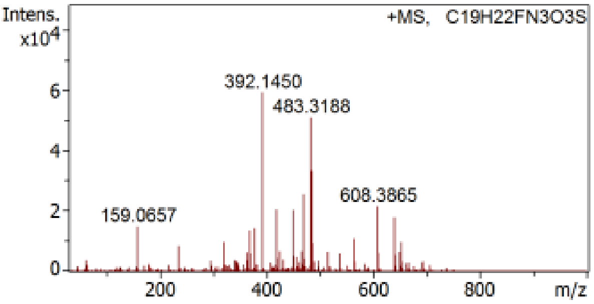

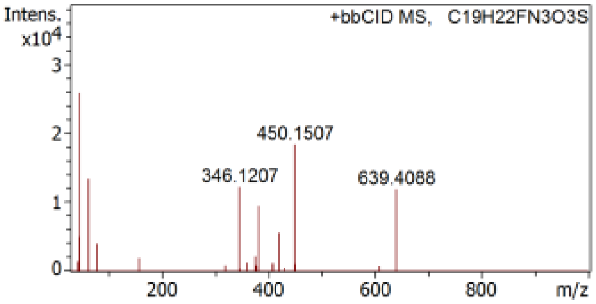

#	m/z	Res.	S/N	I	I %	FWHM
1	159.0657	28975	253.1	14999	25.3	0.0055
2	378.1472	34557	245.1	14524	24.5	0.0109
3	392.1450	43946	999.7	59238	100.0	0.0089
4	418.2881	35421	350.2	20753	35.0	0.0118
5	450.1502	35076	346.1	20508	34.6	0.0128
6	469.3396	33797	432.2	25609	43.2	0.0139
7	483.3188	35297	860.0	50963	86.0	0.0137
8	485.3343	33785	561.5	33275	56.2	0.0144
9	608.3865	34290	366.6	21723	36.7	0.0177
10	639.4086	35502	305.4	18096	30.5	0.0180

#	m/z	Res.	S/N	I	I %	FWHM
1	44.9793	15888	194.9	5062	19.6	0.0028
2	46.9949	16670	995.2	25850	100.0	0.0028
3	63.9978	18777	517.9	13453	52.0	0.0034
4	79.0212	21264	156.3	4061	15.7	0.0037
5	346.1207	32335	473.5	12298	47.6	0.0107
6	376.1138	35141	87.6	2274	8.8	0.0107
7	382.2349	35407	366.4	9516	36.8	0.0108
8	421.2436	33458	218.7	5681	22.0	0.0126
9	450.1507	35681	709.1	18419	71.3	0.0126
10	639.4088	36791	460.3	11957	46.3	0.0174

Figure S6. HRMS spectrum for compound 2.

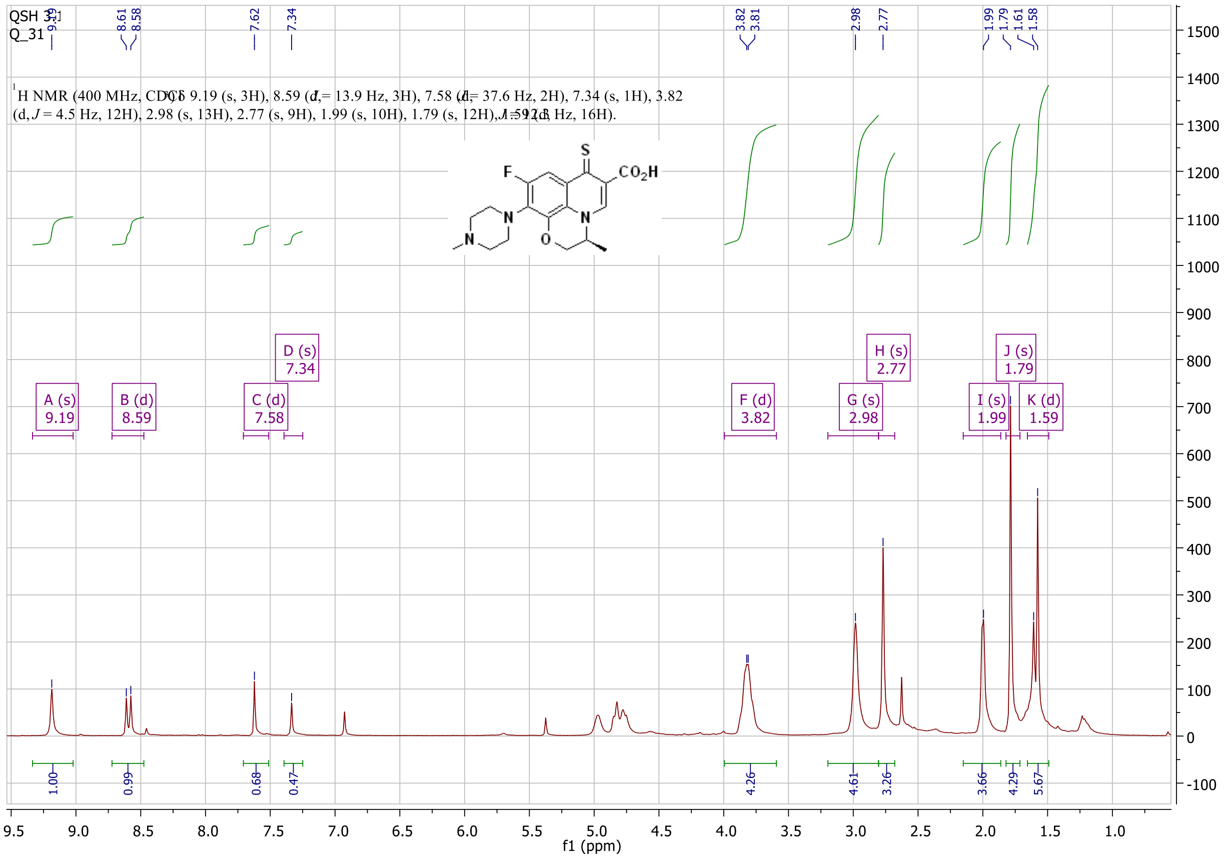

Figure S7. The ^{1}H NMR spectrum for compound 3.

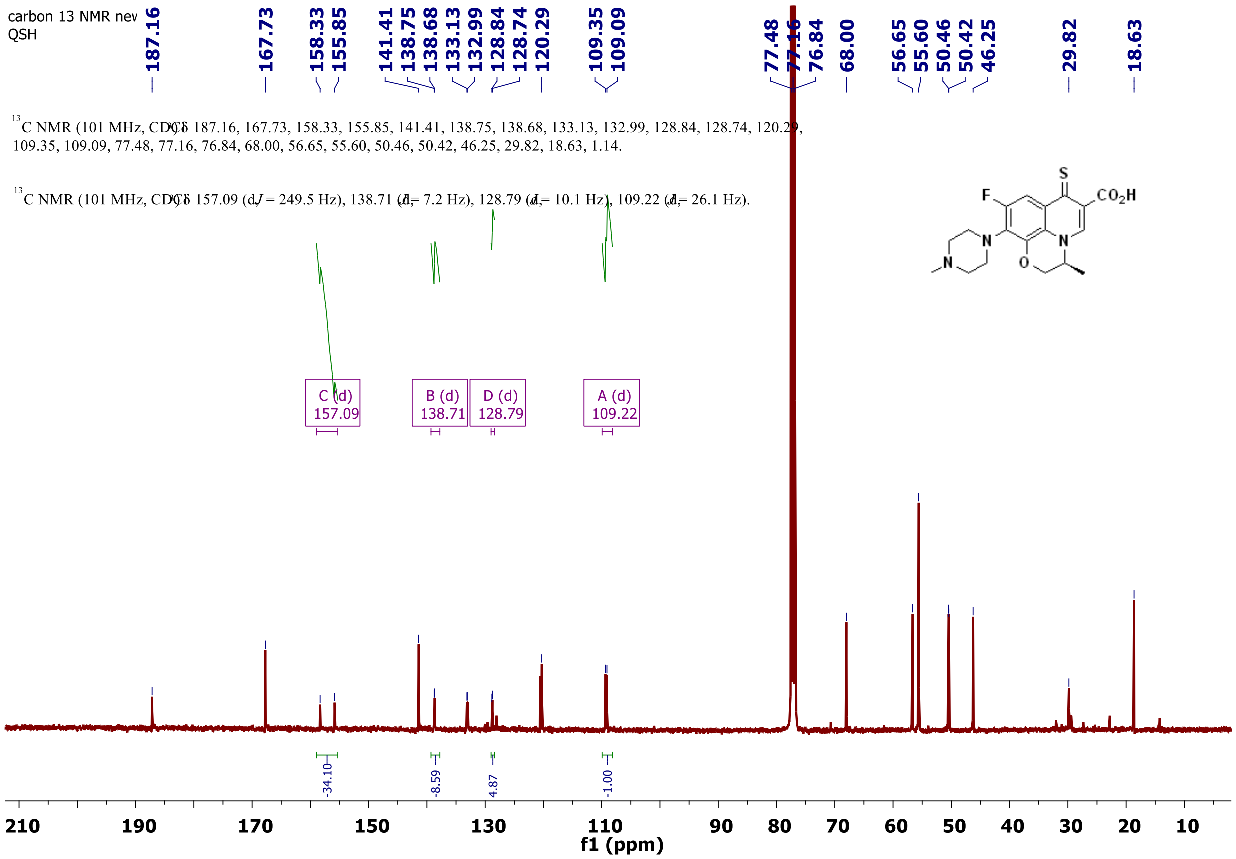

Figure S8. The ^{13}C NMR spectrum for compound 3.

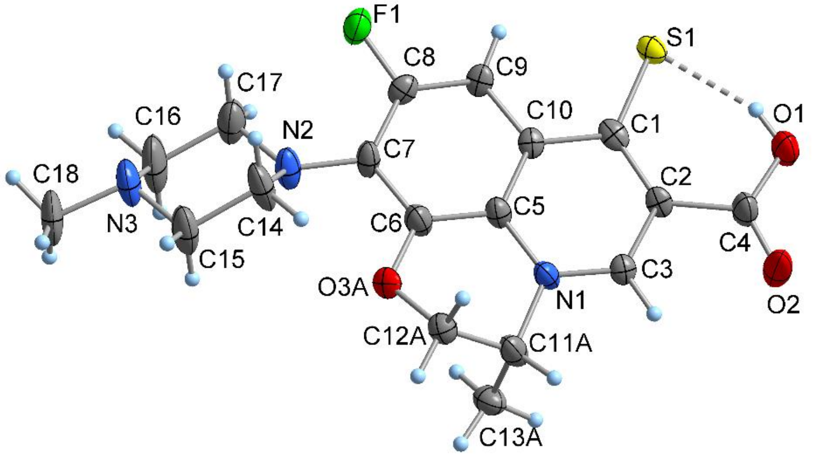

Figure S9. Motif structure of compound 3. Due to the poor quality of the crystal, we could not deposit this compound in the Cambridge database.

Table S1. The zones of inhibition (mm) of the synthesized compounds and levofloxacin against five different bacterial strains. DMSO was used as a negative control.

**Compound**	**Concentration (mM)**	***B. spizizenii***	***S. aureus***	***E. coli***	***P. aeruginosa***	***P. mirabilis***
1	2	19	12	19	NZ	20
	0.5	14	NZ	15	NZ	15
	0.25	NZ	NZ	11	NZ	NZ
	0.125	NZ	NZ	NZ	NZ	NZ
	0.062	NZ	NZ	NZ	NZ	NZ
	0.031	NZ	NZ	NZ	NZ	NZ
	0.015	NZ	NZ	NZ	NZ	NZ
	0.007	NZ	NZ	NZ	NZ	NZ
	0.0039	NZ	NZ	NZ	NZ	NZ
	0.0019	NZ	NZ	NZ	NZ	NZ
	0.0009	NZ	NZ	NZ	NZ	NZ
2	2	20	11	20	NZ	22
	0.5	11	NZ	14	NZ	14
	0.25	NZ	NZ	11	NZ	11
	0.125	NZ	NZ	NZ	NZ	NZ
	0.062	NZ	NZ	NZ	NZ	NZ
	0.031	NZ	NZ	NZ	NZ	NZ
	0.015	NZ	NZ	NZ	NZ	NZ
	0.007	NZ	NZ	NZ	NZ	NZ
	0.0039	NZ	NZ	NZ	NZ	NZ
	0.0019	NZ	NZ	NZ	NZ	NZ
	0.0009	NZ	NZ	NZ	NZ	NZ
3	2	26	21	23	13	25
	0.5	20	15	19	NZ	20
	0.25	16	NZ	16	NZ	19
	0.125	12	NZ	14	NZ	15
	0.062	NZ	NZ	10	NZ	11
	0.031	NZ	NZ	[2] NZ	NZ	NZ
	0.015	NZ	NZ	[2] NZ	NZ	NZ
	0.007	NZ	NZ	[2] NZ	NZ	NZ
	0.0039	NZ	NZ	[2] NZ	NZ	NZ
	0.0019	NZ	NZ	[2] NZ	NZ	NZ
	0.0009	NZ	NZ	[2] NZ	NZ	NZ
Levofloxacin	2	35	32	26	29	29
DMSO	5%	NZ	NZ	NZ	NZ	NZ

NZ**:** No Zone of inhibition.

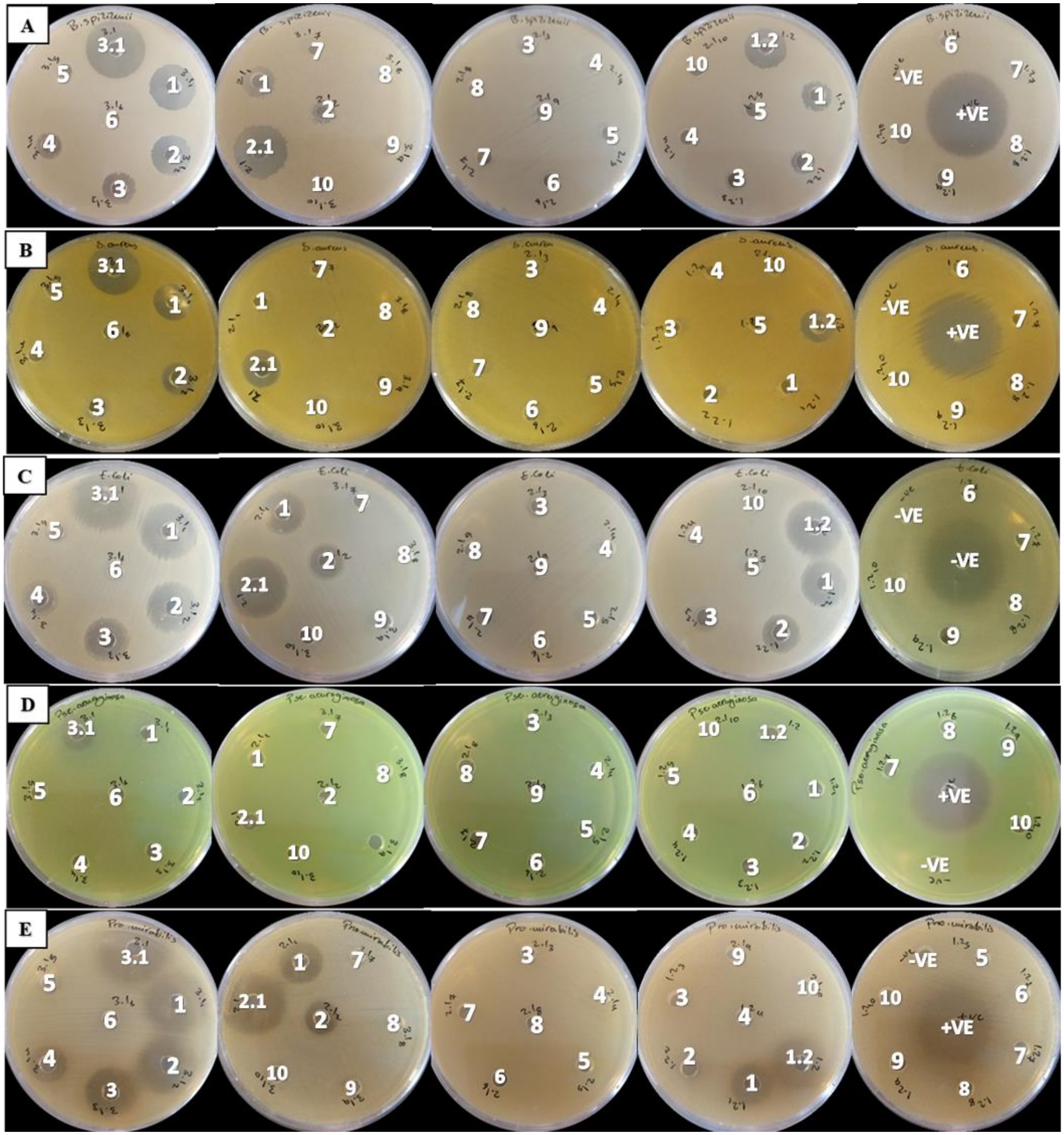

Figure S10. The zones of inhibition for the tested compounds at stock solutions and 0.5 (1), 0.25 (2), 0.125 (3), 0.062 (4), 0.031 (5), 0.015 (6), 0.007 (7), 0.0039 (8), 0.0019 (9) and 0.0009 (10) mM concentration measured by disk diffusion method against: **A.** *Bacillus spizizenii*, **B.** *Staphylococcus aureus*, **C.** *Escherichia coli* (*E. coli*), **D.** *Pseudomonas aeruginosa*, **E.** *Proteus mirabilis*. Levofloxacin was used as the positive control (+VE), DMSO (5%) as negative control (-VE).

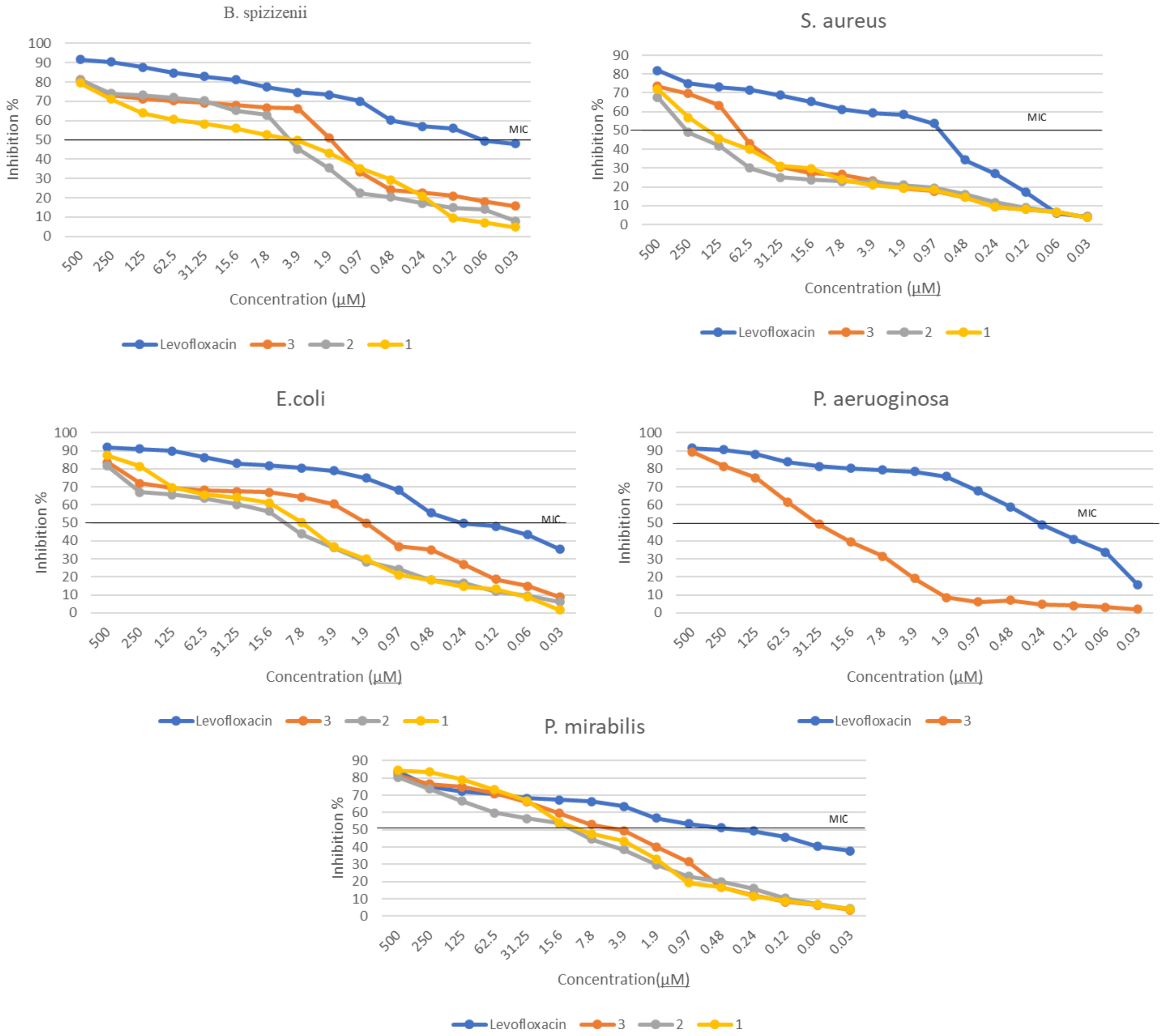

Figure S11. The percentage of inhibition of several bacterial strains by compounds 3 (orange), 2 (gray), 1 (yellow) and levofloxacin (blue).
